# Ingredient Functionality of Soy, Chickpea, and Pea Protein before and after Dry Heat Pretreatment and Low Moisture Extrusion

**DOI:** 10.3390/foods13142168

**Published:** 2024-07-09

**Authors:** Jordan Pennells, Louise Trigona, Hetvi Patel, Danyang Ying

**Affiliations:** 1 CSIRO Agriculture & Food, 671 Sneydes Rd, Werribee, VIC 3030, Australia; jordan.pennells@csiro.au (J.P.); louise.trigona@bordeaux-inp.fr (L.T.); hpat0035@student.monash.edu (H.P.); 2 Department of Food Processing & Biological Engineering, École Nationale Supérieure de Matériaux, d’Agroalimentaire et de Chimie (ENSMAC), University of Bordeaux, 16 Av. Pey Berland, 33600 Pessac, France; 3 Department of Chemical Engineering, Monash University, Wellington Rd, Clayton, VIC 3800, Australia

**Keywords:** twin-screw extrusion, texturized vegetable protein, protein denaturation, ingredient functionality, plant-based meat

## Abstract

This study investigates the impact of dry heat pretreatment on the functionality of soy, chickpea, and pea protein ingredients for use in texturized vegetable protein (TVP) production via low moisture extrusion. The protein powders were heat-treated at temperatures ranging from 80 °C to 160 °C to modulate the extent of protein denaturation and assess their effects on RVA pasting behavior, water absorption capacity (WAC), and color attributes. The results indicate that the pretreatment temperature significantly influenced the proteins’ functional properties, with an optimal temperature of 120 °C enhancing pasting properties and maintaining WAC, while a higher pretreatment temperature of 160 °C led to diminished ingredient functionality. Different protein sources exhibited distinct responses to heat pretreatment. The subsequent extrusion processing revealed significant changes in extrudate density and color, with increased density and darkness observed at higher pretreatment temperatures. This research provides insights into the interplay between protein sources, pretreatment conditions, and extrusion outcomes, highlighting the importance of controlled protein denaturation for developing high-quality, plant-based meat analogues. The findings have broad implications for the optimization of meat analogue manufacturing, with the aim of enhancing the sensory experience and sustainability of plant-based foods.

## 1. Introduction

Global interest in maintaining ethical, sustainable, and healthy diets has driven the development of plant-based food as alternatives to traditional meat products. These products, predominantly made with plant protein-rich ingredients, are typically designed to mimic the sensory and nutritional qualities of animal meat. Extrusion has emerged as a key processing technology to convert protein-rich ingredients into meat analogue products, either through low moisture extrusion (typically < 40% moisture content) to generate texturized vegetable protein (TVP) or high moisture extrusion (typically > 40% moisture content) to generate ‘whole cut’ products [1]. The high-temperature, high-pressure extrusion cooking process applies heat and shear force to transform the material into a functionalized product with desirable taste and texture attributes.

Soybean-derived products, such as soy protein concentrate (SPC) or soy protein isolate (SPI), have been the most common ingredients for commercial plant-based meat analogues. Other common plant protein-rich ingredients include wheat gluten and pea protein isolate [2]. However, a wide range of alternative plant protein sources are emerging for TVP products, such as chickpea, rice, lentil, oat, mung bean, faba bean, lupin, hempseed, canola, potato, corn, and peanut protein [3,4]. The quality of TVP and the subsequent post-processed products, such as burger patties and sausages, is significantly influenced by the intrinsic properties of the protein ingredients used. Protein quality for meat analogue production is closely associated with its functionality, which refers to a range of abilities and behaviors that facilitate the development of a high-quality final product for a particular context or application. These functional abilities may include water absorption, water holding, oil binding, solubility, emulsification, gelation or pasting, foaming, amino acid profile, thermal stability, flavor, and texturization [5]. For meat analogue applications, the key attributes of TVP-based products that enhance the overall sensory experience include taste and aroma, textural properties, juiciness, and appearance.

Protein functionality is highly dependent on the processing methods and conditions used during extraction and isolation. Processing treatments for ingredient preparation and food production cause protein unfolding, denaturation, and aggregation, which can significantly impact functionality. Controlling these phenomena by applying appropriate processing conditions achieves the desired sensory characteristics while limiting extensive protein denaturation, which could impair nutritive value [6]. For instance, producing protein concentrate or isolate ingredients involves extensive processing, typically utilizing wet or dry extraction methods, which can significantly influence protein denaturation and their corresponding functional properties [7,8]. The strategies to limit protein denaturation during ingredient production in commercial facilities include limiting exposure to elevated temperatures, harsh pH, and excessive shear stress [9]. Despite awareness of these effects, balancing the preservation of protein functionality with product safety, shelf life, desired sensory attributes, and techno-economic considerations remains a challenge. For example, Loveday and Halim (2024) demonstrated that laboratory-prepared protein isolates exhibited less protein denaturation and higher solubility compared to commercial isolates [10], highlighting the potential for the refinement of protein extraction and isolation techniques to optimize the functional properties of plant-based protein ingredients for various food applications.

Protein denaturation is often seen as detrimental during protein extraction due to its impact on ingredient solubility and functionality [11]. However, this process becomes essential during extrusion cooking, where controlled denaturation enables protein unfolding, polymerization, and realignment in the material flow direction to form a meat-like fibrous texture [12]. However, excessive denaturation can impact water binding, nutritional value, and texture [13]. Thus, understanding and optimizing protein denaturation is crucial for producing high-quality TVP products. Several questions remain about the impact of protein denaturation on TVP extrusion outcomes, such as those regarding which plant protein sources are more resistant or susceptible to denaturation during extraction and extrusion, the optimal level of denaturation during extrusion, and the factors that promote or prevent denaturation. In addition, there is growing evidence that partial or controlled denaturation may benefit emulsification, gelation, foaming, and texturization properties [14,15,16].

This study investigates dry heat pretreatment as a preprocessing tool to modulate protein denaturation (i.e., ingredient functionality) and improve the physical and textural attributes of extruded TVP products. To induce denaturation of plant protein ingredients, soy, pea, and chickpea protein powders were heated at different temperatures (80 °C, 100 °C, 120 °C, 140 °C, and 160 °C) for 2 h. Various parameters, including pasting properties, water absorption capacity, particle size, DSC, and color were measured to evaluate the impact of pretreatment temperature on ingredient functionality. Subsequently, extrusion was performed on a subset of these samples—raw, 120 °C, and 160 °C—to evaluate the impacts on extrudate properties, such as density, porosity, texture, color, and water-holding capacity. This study offers important insights into protein ingredient functionality and the optimization of meat analogue manufacturing, emphasizing the importance of understanding protein denaturation as a major factor influencing ingredient quality.

## 2. Materials and Methods

### 2.1. Materials

Three protein-rich ingredients were used as inputs into the low moisture extrusion process in this study. The ingredients included soy protein isolate (SPI) obtained from Archer Daniels Midland (Chicago, IL, USA), chickpea protein concentrate (CPC) obtained from Yantai T. Full Biotech Co., Ltd. (Zhaoyuan, China), and yellow pea protein concentrate (YPPC) obtained from Australian Plant Proteins (Melbourne, Australia). The composition of each material was based on the information provided by the supplier, as shown in Table 1.

### 2.2. Experimental Methodology

The experimental design for this study involved two stages of characterization of the ingredients before extrusion and the extruded products, with the ingredients undertaking a 3 × 6 full factorial design for the 3 plant protein ingredients across 6 dry heat pretreatment levels. The extrudates involved a 3 × 3 full factorial design for the 3 plant protein ingredients across a subset of 3 heat pretreatment levels (untreated, 120 °C, and 160 °C); all processing took place under consistent extrusion conditions.

### 2.3. Ingredient Characterization

#### 2.3.1. Heat Pretreatment

All the protein ingredients were pretreated under dry heat conditions (0% relative humidity) in a conventional laboratory oven for 2 h at 5 different temperature levels—80 °C, 100 °C, 120 °C, 140 °C, and 160 °C—in addition to an untreated control sample (raw).

#### 2.3.2. Pasting Properties

To determine the pasting characteristics of the raw and pretreated ingredients, a RVA4800 Rapid Visco Analyzer (PerkinElmer, Waltham, MA, USA) was utilized. For the analysis, 3.5 g of each sample was combined with 25 g of water and premixed by hand to minimize protein clumps and generate a consistent mixture. The pasting profile was initially maintained at a temperature of 50 °C for 60 s, followed by a temperature increase to 140 °C at a rate of 10.7 °C/min, held at 140 °C for 2 min and 30 s, then cooled to 50 °C at the same rate and maintained for an additional 2 min. Peak viscosity, peak viscosity temperature, time to peak viscosity, and end viscosity were measured and recorded during characterization. Each specimen was subjected to this procedure in triplicate.

#### 2.3.3. Water Absorption Capacity

Water absorption capacity (WAC) was conducted according to Webb et al. [17]. In brief, 2.5 g of raw and pretreated powder samples were placed in centrifuge tubes containing 30 mL of deionized water. The samples were vortexed until they were homogenously dispersed and allowed to sit at room temperature for 30 min with two additional mixings in that time. Then, they were centrifuged at 3000× *g* for 30 min. The supernatant was then carefully decanted from the tube, with the weight of the wet sample measured. WAC was calculated based on Equation (1).

(1) $$WAC\;\left(\frac{g\;water}{g\;sample}\right)=\frac{W_{f}-W_{i}}{W_{i}}$$

where $W_{f}$ is the weight of the sediment and $W_{i}$ is the weight of the dry sample.

#### 2.3.4. Particle Size Distribution

The particle size distribution for the raw and pretreated ingredient samples was determined with a series of sieves with varying aperture sizes (0.53 mm, 0.3 mm, 0.18 mm, 0.15 mm, 0.075 mm, and a base catchment), using a vibrating machine at 0.4 amplitude for 2 min to facilitate the separation. Approximately 20 g of sample was placed within the top sieve, with the vibrating separator operating for 2 min at 0.4 amplitude. Subsequently, the contents of each sieve were weighed to enable the determination of the particle size distribution for each sample.

#### 2.3.5. Color

The color of the raw, pretreated, and ground extrudate samples was quantified using a handheld Minolta CR-300 Chroma meter (Konica Minolta Sensing Americas, Ramsey, NJ, USA), which provided data for the CIELAB values of black/white lightness (L*), red/green (a*), and yellow/blue (b*) color coordinates. The powders were measured in a plastic pouch with at least 5 mm thickness of the sample for each measurement. The total color difference (Δ*E*) was calculated according to Equation (2):

(2) $$\Delta E=\sqrt{{\left(L_{P}-L_{R}\right)}^{2}+{\left(a_{P}-a_{R}\right)}^{2}+{\left(b_{P}-b_{R}\right)}^{2}}$$

where *P* refers to the heat-pretreated ingredient sample, and *R* refers to the raw ingredient sample.

#### 2.3.6. DSC

The thermal properties were measured by differential scanning calorimetry (DSC 1, Mettler Toledo, Melbourne, Australia). The instrument was calibrated using water, indium, and zinc. The protein powders were initially hydrated to a moisture content of ~70% *w*/*w* (±1.0% *w*/*w*) through manual mixing with deionized water and rested for 1 h prior to testing. Samples of ~20 mg (±2.0 mg) were weighed into 40 μL aluminum crucibles and hermetically sealed. The samples were heated from 20 to 120 °C at a rate of 5 °C/min, with an empty sealed pan used as a reference. Data were analyzed via the STARe version 12.1 software.

### 2.4. Extrusion Processing

A laboratory scale, co-rotating and intermeshed twin-screw extruder (KDT30-II, Jinan Kredit Machinery Co., Ltd., Jinan, China) was used for TVP production. The extruder configuration involved a 30 mm screw diameter, a 20:1 screw length/diameter ratio, and a circular die with a 2 mm diameter. The barrel was segmented into a feeding zone and four zones with temperature control, which were heated by an electric cartridge heating system and cooled with town water. The temperature and screw speed were regulated with a control panel, where the extruder responses, such as the motor electric current and die pressure, were shown. The extruder barrel temperatures from the feed port to the die were set at 25 °C, 50 °C, 145 °C, and 130 °C, and were kept constant during processing. The extrusion operation was set at a feed rate of 4.8 kg/h and a screw speed of 460 rpm. Throughout all the experimental runs, the in-barrel moisture content was maintained at 36%, which was controlled by adjusting the water feed rate with a FEDOS E/DX8 piston pump (Process Pumps Pty. Ltd., Melbourne, Australia), which was calibrated before the extrusion run. The water port was located at around two-thirds of the screw length, closer to the powder feed end. The extruded samples were collected for physicochemical analysis, when the process conditions reached a steady state, as indicated by a relatively constant value for die pressure and material throughput. The samples were collected and immediately dried for 2–3 days in a batch oven at 60 °C prior to analysis.

### 2.5. Extrudate Characterization

#### 2.5.1. Physical Properties

The true density of the TVP extrudates was measured in accordance with the method outlined by Lee et al. [18]. In brief, a random selection of extrudate pieces were sampled and ground using benchtop grinding (GM2000, MEP Instruments, Melbourne, Australia) at 4000 rpm for 30 s, before being transferred into a 5 mL graduated cylinder. The cylinder was tapped gently against the bench approximately 50 times to allow the powder to settle. The mass of the powder was weighed, and this value was divided by the corresponding volume, as per the calculation demonstrated in Equation (3).

(3) $$\rho_{\mathrm{true}}\left(\frac{g}{L}\right)=\frac{m_{powder}}{V_{powder}}$$

The apparent density was measured in accordance with the method outlined by Martin et al., with minor modifications [19]. Initially, 50 cm^3^ of cornmeal was placed into a 100 mL volumetric cylinder. Pieces of extrudate with a total weight of approximately 2 g were then added to the cylinder containing the cornmeal and weighed. The apparent density was calculated by dividing the mass of the extrudate pieces by the volume of cornmeal displaced within the cylinder, as demonstrated in Equation (4).

(4) $$\rho_{\mathrm{apparent}}\left(\frac{g}{L}\right)=\frac{m_{extrudate}}{V_{final}-V_{initial}}$$

where $V_{final}$ is the final volume of the cornmeal and extrudate mixture in the cylinder, and $\;V_{initial}$ is the initial volume of the cornmeal in the cylinder before the addition of the extrudate. Finally, porosity was calculated based on the difference between the apparent and true densities, expressed as a fraction of the true density based on Equation (5).

(5) $$\phi \left(\%\right)=\frac{\rho_{\mathrm{true}\;}-\rho_{\mathrm{apparent}}}{\rho_{\mathrm{true}}}$$

#### 2.5.2. Textural Properties

The textural properties of the extrudates were evaluated in accordance with the methodology described by Kaleda et al. [20]. In brief, the dry extrudates were initially rehydrated in tap water at a temperature of 60 °C for a duration of 7 min, with excess water removed by letting the samples sit on a paper towel. Subsequently, the rehydrated extrudates were subjected to a double compression test using a TA-XT2 Texture Analyzer (Stable Micro Systems, Surrey, UK), with a 35 mm diameter flat plate probe (P/35). The conditions were 75% compression of their original thickness, a probe speed of 1 mm/s, and an intermission of 2 s between compressions. The textural properties focused on in this study included hardness, springiness, and chewiness. The quantitative results for the textural properties were determined using the software integrated with the texture analyzer, with calculations shown in Equations (6)–(8).

(6) $$Hardness\;\left(g\right)=Peak\;force\;of\;the\;first\;compression$$

(7) $$\mathrm{Springiness}\left(-\right)=\frac{Recovery\;distance\;between\;first\;\&\;second\;compression}{Original\;compression\;distance}$$

(8) $$Chewiness\left(g\right)=\mathrm{Hardness}\times \mathrm{Cohesiveness}\times \mathrm{Springiness}$$

#### 2.5.3. Water-Holding Capacity

The water-holding capacity (WHC) of the extrudates was conducted according to Webb et al., with a slight modification [17]. Approximately 10 g of both whole and ground extrudates were immersed in excess water at ambient temperature for 20 min, then drained over a 73 μm mesh screen for 5 min. WHC was then calculated as the ratio of the retained water compared to the initial mass of the sample, according to Equation (9):

(9) $$\mathrm{WHC}\left(\%\right)=\frac{m_{\mathrm{water}\;}+m_{\mathrm{extrudate}}}{m_{\mathrm{extrudate}}}\times 100$$

### 2.6. Statistical Methodology

#### 2.6.1. Normality Testing

The Shapiro–Wilk test was used to assess the normality of the data distribution for each material property. A significance level of α = 0.05 was used to determine normality.

#### 2.6.2. Analysis of Variance (ANOVA)

A one-way ANOVA was conducted for the normally distributed data to evaluate the statistical significance of the differences between the protein source or pretreatment groups. A post hoc Tukey’s honestly significant difference (HSD) test was used for pairwise comparisons when significant differences were detected.

#### 2.6.3. Kruskal–Wallis Test

For data that did not pass the normality test, the non-parametric Kruskal–Wallis test was used to evaluate the statistical significance between groups. A post hoc Dunn’s test with Bonferroni correction was applied for pairwise comparisons when significant differences were identified.

#### 2.6.4. Partial Least Squares Regression

Partial least squares (PLS) regression was used to investigate the relationship between the properties of powder ingredients and various attributes of the extrudates. The analysis was performed using the *pls* package in R, with preprocessing of raw data and cross-validation to prevent overfitting. The predictor variables (X) included the measured powder properties, while the response variables (Y) encompassed a range of textural and other extrudate properties. Detailed descriptions of the PLS methodology can be found in the Supplementary Materials.

#### 2.6.5. Monte Carlo Simulation

Monte Carlo simulations (*n* = 100) were conducted to account for variability and to evaluate the robustness of the model predictions against random sampling errors. This involved repeated random subsampling and modeling to estimate the stability of the PLS coefficients across iterations. Summary statistics and confidence intervals were calculated to assess the precision of the estimates.

## 3. Results and Discussion

### 3.1. Ingredient Functionality

#### 3.1.1. Pasting Properties

The RVA is a tool commonly applied to the physiochemical characterization of cereal starches but can also be used for protein-rich ingredients [21,22]. Understanding the pasting properties of protein ingredients provides insights into the processability and quality potential of TVP products. The rheological characteristics of the protein mixture directly influences the textural qualities and functional performance of the extruded TVP. An RVA was used in this study to evaluate the impact of a heat pretreatment on the functionality of different protein-rich ingredients.

**Figure 1:**
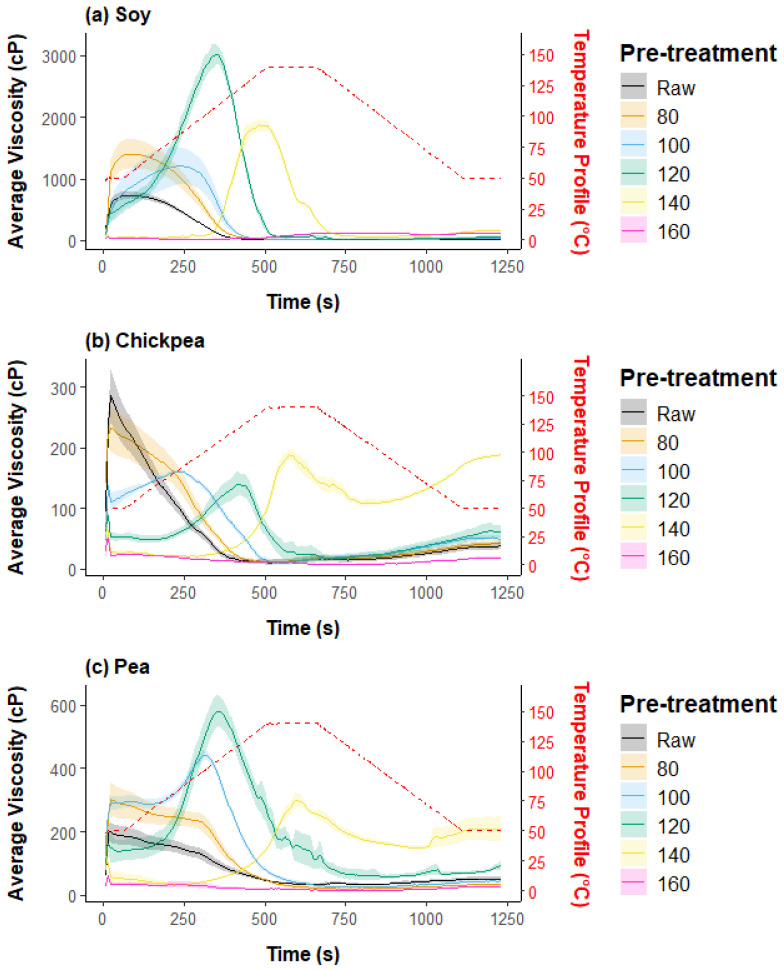
Impact of heat pretreatment temperature on the RVA pasting profile of (**a**) soy, (**b**) chickpea, and (**c**) pea protein ingredients.

Figure 1 outlines the combined pasting profiles of both the raw and heat-pretreated samples for the three different plant protein ingredients. The solid line represents the average profile of three replicates for each sample, while the shadow represents the standard error of the viscosity profiles. The dashed red line indicates the temperature of the pasting profile.

It is clear that the pasting properties are influenced by the temperature of the heat pretreatment applied to each type of protein. For the soy protein (Figure 1a), there is an observable increase in peak viscosity with heat treatment at 80 °C and 100 °C compared to the raw sample baseline, with the peak shifting to higher viscosities at these temperatures. Notably, the sample treated at 120 °C exhibits a substantial rise in peak viscosity, reaching the maximum value among the treatments at approximately 3000 cP. This suggests an enhancement of protein gelatinization and potential protein aggregation after this pretreatment temperature. The mechanism behind this result is likely related to the denaturation of the native proteins when the temperature is increased above 75 °C, causing protein solubility to decrease and resulting in increased viscosity [23]. The gelling properties of the ingredients depend not only on the total protein content but also on the plant origin, the denaturation and aggregation state of the proteins, and the interaction with non-protein components [24]. However, as the pretreatment temperature increases to 140 °C and above, there is a notable decrease in the peak viscosity, with a complete absence of viscosity build-up for the 160 °C pretreated sample. This suggests a changing degree of protein denaturation as a function of pretreatment temperature and complete loss of functionality above a particular pretreatment temperature threshold, with the altered protein structure limiting the viscous properties of the ingredient.

This non-linear relationship between pretreatment extent and ingredient functionality was also observed in a previous study by Jiang et. al., where a 90 s microwave treatment of raw faba beans generated a peak viscosity of 800 cP compared to no peak for the untreated sample, with the 4 min microwave treatment resulting in a final viscosity that was one tenth of the untreated sample [14]. The authors identified the fact that there is a surprising lack of literature on the impact of dry heating pretreatment on the pasting properties of legume flours.

For the chickpea protein samples in Figure 1b, there is a complete loss of viscosity build-up for the 160 °C pretreatment level. However, key differences with this sample include a viscosity spike at the start of the pasting profile for all samples, to varying degrees, which may be attributed to the difficulty involved in creating a consistent mixture for these samples, especially for the raw and 80 °C pretreated samples. Another key difference is that the 140 °C pretreated sample showed the highest peak viscosity for the chickpea sample (when disregarding the initial viscosity spike), which indicates that different protein types react differently to heat pretreatment conditions.

For the yellow pea protein samples in Figure 1c, peak viscosity is achieved again for the 120 °C pretreatment level, with a substantial drop in peak viscosity for the higher temperature levels. It once again appears that some degree of heat pretreatment enables a viscosity increase over the pasting profile, but in a non-linear manner that leads to a drop in peak viscosity at a particular pretreatment temperature. Notably, the peak viscosity observed for chickpea and pea protein is markedly lower than that for soy protein, which is approximately 300 cP and 600 cP, respectively, compared to approximately 3000 cP for soy. This indicates a tenfold difference in peak viscosity when comparing chickpea to soy, and a fivefold difference when comparing pea to soy.

Lastly, the observed decrease in viscosity peak before the temperature reaches its maximum during RVA analysis may be attributed to multiple factors. Protein denaturation and aggregation initially increase viscosity as protein networks form, but beyond a certain temperature, these aggregates may become more compact, leading to a decrease in viscosity. Shear stress applied during the RVA measurement can further contribute to protein unfolding and the breakdown of weaker protein aggregates. Additionally, the gel-like network formed by the protein aggregation might begin to break down under the combined effects of temperature and shear. Another factor to consider is the potential sticking of the thickened gel to the walls of the RVA cup, reducing the interaction with the stirrer and thus decreasing the measured torque, which is directly related to the measured viscosity.

#### 3.1.2. Water Absorption Capacity

Water absorption capacity (WAC) measures the ability of a material to absorb and retain moisture under a specific force, such as centrifugation, which is important for assessing the functionality and quality of protein ingredients in TVP products. WAC is influenced by the hydrophilic characteristics of protein and carbohydrate components, the source of the plant protein, the processing conditions during protein extraction, and the particle size of the powder [25,26,27].

**Figure 2:**
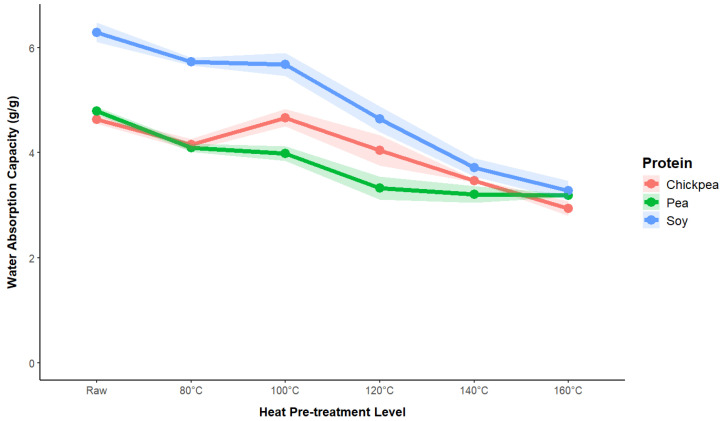
Impact of heat pretreatment temperature on water absorption capacity in soy, pea, and chickpea protein ingredients.

A comparative analysis of WAC for chickpea, pea, and soy proteins subjected to various levels of heat pretreatment, as presented in Figure 2, demonstrates a decreasing trend with a higher pretreatment temperature for all the protein sources. This reduction across all the proteins indicates that the structural changes induced by higher temperatures significantly impair the hydration properties of the protein. This result is in contrast with those of previous studies, where a higher degree of protein denaturation resulted in enhanced water-trapping behavior [28,29]. It is hypothesized that the dominant mechanism in this experiment is the denaturation and unfolding of protein molecules. This process leads to the exposure of hydrophobic groups previously hidden within the tertiary structure of the protein, thereby reducing the affinity of the sample for water. Heating can disrupt hydrogen bonds and ionic interactions, which are crucial for water retention, resulting in protein denaturation and aggregation. These structural changes expose hydrophobic regions and reduce water-holding capacity (WHC) and can expel water through syneresis. Additionally, Maillard reactions between proteins and sugars during heating can form advanced glycation end products, further decreasing water absorption capacity (WAC). The combined effects of protein aggregation and structural changes lead to a more dense and tightly packed pellet structure after centrifugation, excluding water.

With regard to the protein source, soy maintains the highest WAC across all treatment levels, in addition to starting with a significantly higher value for the raw protein sample. This result is in line with past studies that have compared WAC values across multiple protein sources [20,30,31].

#### 3.1.3. Particle Size Distribution

**Figure 3:**
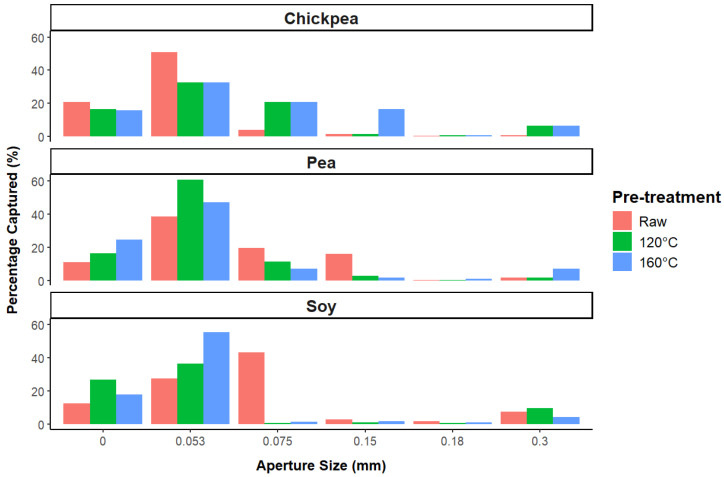
Impact of heat pretreatment temperature on particle size distribution of soy, pea, and chickpea protein ingredients.

To evaluate the hypothesis presented based on the WAC measurements, the particle size distribution of a subset of the ingredient samples (raw, 120 °C, and 160 °C for chickpea, pea, and soy) was investigated and is presented in Figure 3. Comparing the particle size of the chickpea samples, it is evident that the pretreated samples at 120 °C and 160 °C have a larger particle size than the raw samples. Almost 20% of the particles treated at 120 °C and 160 °C are between 0.053 mm and 0.075 mm, whereas in the raw sample, less than 10% of the particles exceed 0.053 mm. Additionally, it is noticeable that in the sample treated at 160 °C almost 20% of the particles are still between 0.075 mm and 0.15 mm. Therefore, for chickpea proteins, it can be concluded that particle size increases with the level of heat pretreatment. However, for the pea and soy proteins, it is observed that the particle size decreases with the heat pretreatment level. Looking at the pea protein, the raw sample has 40% of its particles above 0.075 mm and still has 20% above 0.15 mm, while the samples treated at 120 °C and 160 °C have less than 20% of their particles above 0.075 mm. A similar trend is observed for soy protein, where the raw sample has more than 50% of its particles above 0.075 mm, while the treated samples have less than 10% above 0.75 mm. In conclusion, these observations indicate that the heat treatment level does not have the same impact on the particle size of the different proteins. Moreover, the particle size decreases with the heat treatment level for the pea and soy proteins. This challenges the initial hypothesis that the decrease in WAC is solely due to an increase in particle size. While this may hold true for chickpea protein, it does not seem applicable to soy and pea proteins. Therefore, it could be speculated that the decrease in WAC is not related to particle-scale aggregation but may be due to a loss in functionality from protein denaturation, leading to molecular-level aggregation that can adversely affect the water absorption capacity of the protein powder [14].

#### 3.1.4. Powder Color

**Figure 4:**
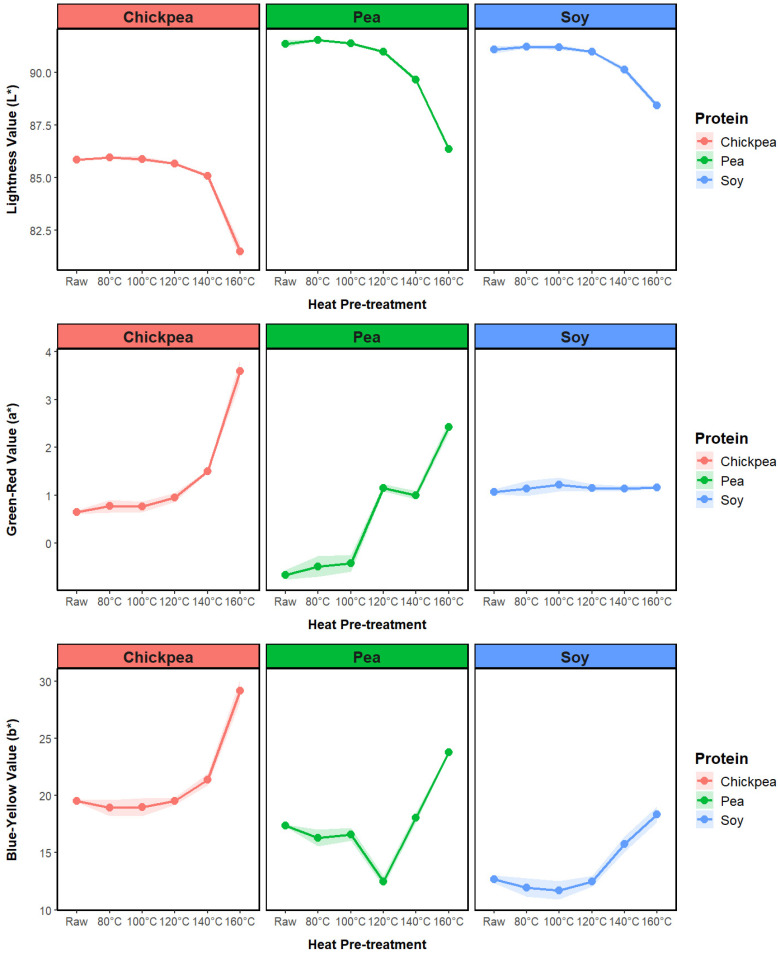
Impact of heat pretreatment temperature on the color values of soy, pea, and chickpea protein ingredients.

Following the application of heat treatment, a discernible browning effect was observed in the ingredients. To quantitatively assess this change in color, an analytical procedure was implemented, with the analysis of the results focusing on the lightness (L*) parameter (Figure 4). While chickpea protein is naturally darker than soy and pea protein, the data showed a consistent trend across all three types. The lightness values remained similar for the raw samples and those treated at 80 °C, 100 °C, 120 °C, and 140 °C. However, a noticeable decrease in lightness occurred beyond the 160 °C heat treatment. This change is likely due to the Maillard reaction, which is a chemical reaction process between amino acids and reducing sugars that leads to browning and typically occurs due to cooking at elevated temperatures [32]. The drop in lightness was slightly less noticeable in the soy protein samples. This may be due to the soy protein isolate having a higher protein concentration, resulting in a lower concentration of reducing sugars available for the Maillard reaction. The Maillard reaction can also impact protein functionality, which could be an explanation for the results of the RVA pasting profile and WAC at the highest pretreatment level [32]. This relationship was also observed for the total color difference between the raw and pretreated powder samples (Table 2).

#### 3.1.5. DSC

Thermal analysis conducted through DSC was performed to understand the denaturation behavior of the three protein powders that were pretreated under different conditions, as shown in Table 3. First, it was found that soy protein isolate did not exhibit a protein denaturation peak, regardless of whether the sample was in the raw or heat pretreated state. On the other hand, chickpea protein concentrates exhibited denaturation peaks for all the samples tested, with a lower magnitude of denaturation enthalpy (ΔH) from the raw sample (−0.25 J/g) to the 160 °C pretreated sample (−0.07 J/g). Interestingly, pea protein concentrate exhibited the highest magnitude of denaturation enthalpy for the raw (−0.71 J/g) and 120 °C pretreated (−0.60 J/g) samples but lost its denaturation peak after the 160 °C pretreatment level. This result indicates that a significant transformation of the protein structure occurred between the 120 °C and 160 °C heat pretreatment levels, which fits with previous results for the substantial shift in protein powder color and RVA pasting properties. In terms of other differences between the protein powders, the chickpea protein exhibited a significantly higher onset temperature (89.5–97.8 °C) and peak temperature (102.9–107.1 °C) for protein denaturation, compared to the pea protein onset temperature (78.0–79.9 °C) and peak temperature (85.9–87.8 °C). It is important to consider that different protein fractions, such as globulins and albumins in chickpea and pea proteins, and glycinin and β-conglycinin in soy proteins, exhibit distinct denaturation temperatures and contribute variably to functionality. Future analysis should focus on identifying these specific fractions to better understand their thermal behavior.

The DSC results of onset temperature, peak temperature, and denaturation enthalpy are highly dependent on the moisture content at which the protein powder was prepared. In this study, the protein samples had their moisture content adjusted to approximately 70% *w*/*w*, which generated sticky paste-like substances in most cases. Previous studies have performed DSC analysis at a moisture content ranging between dry powders at a low value of ~8%, up to 90% moisture content. Tang et al. found that various dry protein powder samples heated at a rate of 10 °C/min exhibited a peak denaturation temperature ranging between 141 °C and 169 °C [33]. Ricci et al. found that the denaturation peak temperatures for various legume protein isolate samples conditioned at a 15% moisture content were within a high temperature range from 183 °C to 212 °C, without any correlation to protein purity or plant origin [34]. However, for a moisture content more closely related to the current study, Osen et al. uncovered a protein denaturation peak temperature of 89.1 °C for a commercial pea protein isolate prepared to ~70% moisture content [23], while Cai et al. demonstrated peak denaturation temperatures of various plant protein samples at a ~70% moisture content that ranged from 86.6 °C to 109.2 °C [35]. In addition, Lefèvre et al. performed DSC analysis on the protein-enriched fraction of various legumes (chickpea, green lentil, and navy bean) at an 80% moisture content, finding that when the samples were preheated to temperatures exceeding the denaturation temperature of a specific protein transition, the corresponding peak disappeared in the DSC thermograms [36]. For example, after a pretreatment of 110 °C, the first denaturation peak for vicilin protein in lentils and chickpeas disappeared, and all the protein denaturation peaks disappeared after a pretreatment of 160 °C, which provides more evidence supporting the theory that a significant, irreversible shift in protein structure occurs for a preheating treatment between 110 °C and 160 °C [36].

The DSC analysis outcomes, with complete thermograph diagrams for all the protein samples presented in the Supplementary Materials, emphasize that there is a considerable extent of denaturation for the commercial protein powders before any heat pretreatment, especially for soy and chickpea protein samples. In this study, the pretreatment temperature level had an impact on the degree of protein denaturation for chickpea and pea protein concentrate, with the major impact occurring below 120 °C for chickpea and between 120 °C and 160 °C for yellow pea. This result aligns with previous literature studies on DSC thermal analysis of plant protein samples. For instance, Sirtori et al. analyzed the level of protein denaturation for pea protein before and after various pretreatment types, including humid heating, dry heating, and mechanical treatment with an Ultraturrax macerator. Their results highlighted that both humid and dry heat pretreatment significantly reduced protein denaturation enthalpy, with reductions ranging from 35.4% to 69.2%, while mechanical pretreatment led to only minor reductions in denaturation enthalpy, ranging from 3.1% to 7.7% [6].

In addition to the moisture content, the DSC heating rate and aspects of the preparation method such as the hydration time can have an impact on the thermal stability behavior [37]. Loveday and Halim (2024) demonstrated that protein powder samples left to hydrate overnight exhibited a reduction in peak temperature and a substantial increase in the size of the protein denaturation endotherms, compared to freshly prepared suspensions [10]. The relatively short hydration time of one hour in this study may result in an overestimation of the peak denaturation temperature and an underestimation of the protein denaturation enthalpy.

### 3.2. Extrusion Outcomes

#### 3.2.1. Visual Appearance

Following ingredient characterization, a subset of samples were selected for extrusion processing to form TVP products, which included the three protein sources across three pretreatment levels—raw (untreated), 120 °C, and 160 °C. The raw samples were selected as a form of control, while the 120 °C level demonstrated the most interesting pasting properties in Figure 1 and the 160 °C level to investigate the impact of functionality loss on extrusion outcomes.

**Figure 5:**
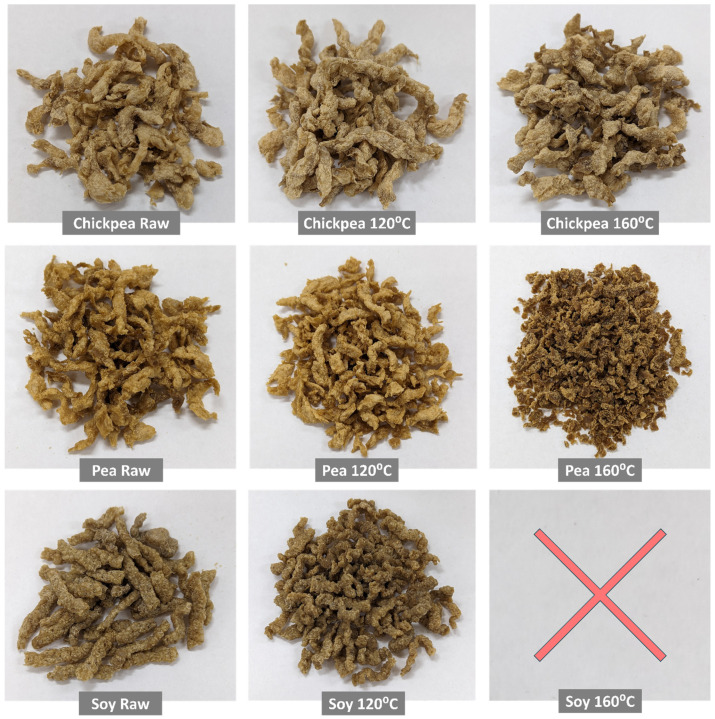
Visual appearance of soy, pea, and chickpea TVP extrudates after various pretreatment conditions.

During the experimental trial, difficulties were encountered in the extrusion of soy protein under the selected operational parameters, especially for the 160 °C pretreated sample, which could not be extruded under the selected processing conditions. The considerably higher maximum peak viscosity of soy protein during RVA characterization, which was approximately 5 times higher than pea protein and 10 times higher than chickpea protein, led to material build-up and backflow within the extruder. This issue manifested as a high and unstable die pressure and as damp protein material accumulating in the feed zone, which caused powder flooding at the feed port. While the decision to standardize the extrusion process conditions aimed to reduce variability in the experimental design and ensure comparability of results, this approach inadvertently introduced a bias in the extrusion outcomes based on the processing conditions selected. The results were then related to the suitability of the extrusion parameters for the specific protein source, rather than to a comparison of the inherent differences between the proteins. With this caveat in mind, the consistent set of extrusion conditions, which were selected based on previous experience with the current extrusion equipment, appeared to create a bias against the soy protein sample. As seen in Figure 5, the soy extrudates were darker and less expanded than the other extrudates. This was also the case for pea 160 °C extrudates and for some pieces of the chickpea 160 °C extrudates. Comparing protein types, the chickpea extrudates show less color change and maintained more of their expansion and structure at higher temperatures than pea and soy, suggesting that different protein sources respond uniquely to heat treatment.

#### 3.2.2. Extrudate Color

**Figure 6:**
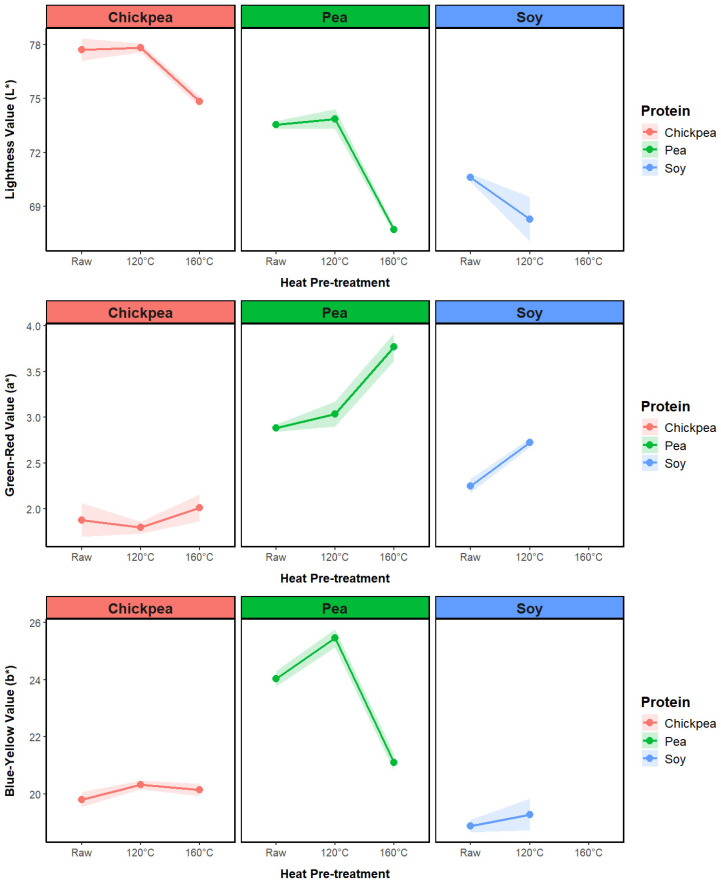
Impact of pretreatment temperature on the color values of soy, pea, and chickpea extrudates.

To quantitatively evaluate color differences between extrudates, the evolution in the CEILAB color values for each protein source is presented in Figure 6. For the pea and chickpea samples, a significant drop in lightness is observed for the 160 °C treated samples. While the drop in lightness for the protein powders with increasing treatment temperature (Figure 4) was directly attributed to Maillard reaction browning and protein denaturation, the color change in the extrudate pieces was more likely due to the lower porosity, which was linked to protein denaturation and loss in ingredient functionality after high-temperature pretreatment. The smoother, less porous surface of the high-temperature samples gave a darker, glossier appearance to the extrudates. In addition, the lower light-scattering ability of the high-temperature-treated samples due to a denser structure generated a darker appearance.

Comparing these results with the powder color values in Figure 4, there is a substantial reduction in lightness following extrusion, ranging from 6.64 for chickpea 160 °C to 22.7 for soy 120 °C. Extrusion cooking can induce the Maillard reaction between sugars and amino acids, leading to protein glycation and the alteration of protein properties [38]. Chickpea had the lowest total difference in color between the powder and extrudate samples (Table 4), which was linked to the ability of the chickpea protein to generate an expanded product across the three pretreatment levels.

#### 3.2.3. Physical Properties

**Figure 7:**
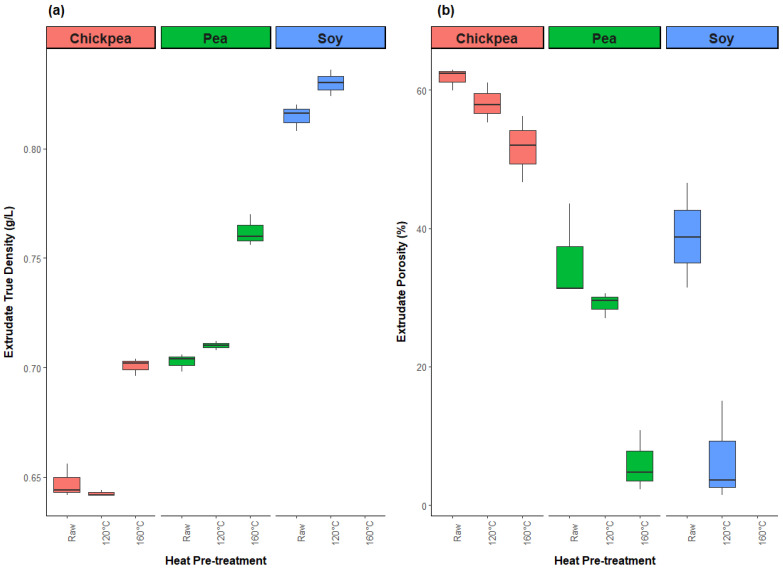
Impact of heat pretreatment temperature on the density (**a**) and porosity (**b**) of soy, pea, and chickpea extrudates.

The true density of extrudate samples ranged from 0.64 g/L for raw chickpea to 0.84 g/L for soy 120 °C, with both soy samples being significantly higher than all the other samples, as shown in Figure 7. As true density provides insight into the properties of the material itself, not just its bulk structure, this result affirms the idea that the highly viscous soy protein resulted in overcooking of the material due to viscous heat dissipation from resistance to flow through the extruder, which caused further protein denaturation and produced a glassy extrudate product that was unable to expand after exiting the die. Another notable result for true density is the substantial increase between the 120 °C and 160 °C pretreatment levels for pea and chickpea, which confirms the reduction in ingredient functionality for the 160 °C temperature level.

This phenomenon is further confirmed by the porosity results, where the pea 160 °C extrudates have an average porosity of only 6.0%, compared to 61.7% for the raw chickpea extrudates. In general, the extrudates become less porous with higher pretreatment temperature. Interestingly, the chickpea samples demonstrate a relatively high porosity, even for the 160 °C temperature level, which indicates that this protein source was able to maintain expansion behavior under the selected extrusion conditions despite the hypothesized ingredient functionality loss. In this study, porosity was investigated as the indicator of product expansion behavior, as the TVP expansion degree was difficult to directly measure due to the irregular shape of the extrudates produced from these protein sources. Similarly, apparent density is the inverse to porosity, and the results were not presented.

#### 3.2.4. Water-Holding Capacity

WHC is an important quality index for TVP products, contributing to the juiciness of the rehydrated TVP product and limiting moisture loss during cooking [18]. WHC is strongly associated with the extrudate porosity, where higher pore volume allows more water to diffuse into the sample and form hydrogen bonds with protein hydroxyl groups [18,39]. This relationship was also observed in the current study, with the chickpea extrudates exhibiting both the highest porosity and WHC, in addition to a general reduction in porosity and WHC with a higher pretreatment temperature. However, an excessively high porosity and WHC may have a detrimental effect on product quality, as a high degree of moisture retained in the product can contribute to a soft and mushy texture [40].

**Figure 8:**
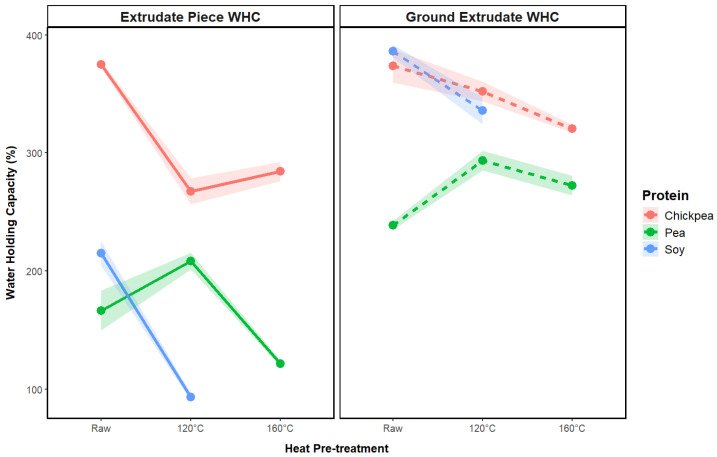
Impact of heat pretreatment temperature on water-holding capacity of soy, pea, and chickpea extrudate pieces and ground extrudate.

Interestingly, as shown in Figure 8, the pea 120 °C sample exhibited a higher WHC (209%) compared to the untreated sample (167%), despite having a similar porosity, with this sample also demonstrating distinct textural properties of lower hardness and higher springiness compared to the other pea samples. These values are approximately in line with previous literature results for pea protein powder extruded under low moisture conditions (30% *w*/*w*), with a WHC of 260% for the whole extrudate pieces [41]. The whole extrudate pieces of untreated chickpea protein exhibited the highest WHC of 375%, while soy 120 °C exhibited the lowest WHC of 93%.

The WHC of the ground samples was predominantly higher than that of the whole extrudate pieces, which is attributed to the higher surface area generated after grinding [42]. The soy samples exhibited a significantly higher WHC in this case, which was most likely due to the high brittleness of these samples in their dry form, which yielded a relatively higher surface area powder after grinding compared to the other two protein sources, enhancing water-binding capabilities.

#### 3.2.5. Textural Properties

The textural properties of extruded TVP were evaluated with a quantitative texture analyzer using the double compression methodology. The key texture metrics include hardness, springiness, and chewiness, which provide insights into the firmness, structural integrity, and elasticity of the product, which are crucial for mimicking meat-like textures. Hardness measures the peak force during the first compression cycle, reflecting resistance to compression. Springiness indicates the ability of the product to recover its shape after deformation, mimicking the semi-elastic and fibrous nature of muscle meat. Chewiness represents the energy required to masticate the product until it is ready for swallowing, combining hardness, cohesiveness, and springiness.

Determining the optimal texture profile of extruded TVP is challenging, as these characteristics can vary widely depending on the intended application and consumer preferences. There is no singular ideal texture profile; individual preferences vary between a firmer and chewier bite for beef analogues versus a softer and juicer bite for chicken analogues. Additionally, the dimensions of the extrudate sample significantly influence the textural properties, necessitating careful sampling for accurate results. This study faced challenges in consistency due to variability in piece morphology among the different protein sources and pretreatment levels, as reflected in the high standard error across the texture metrics, even though 10 extrudate pieces were tested per sample.

**Figure 9:**
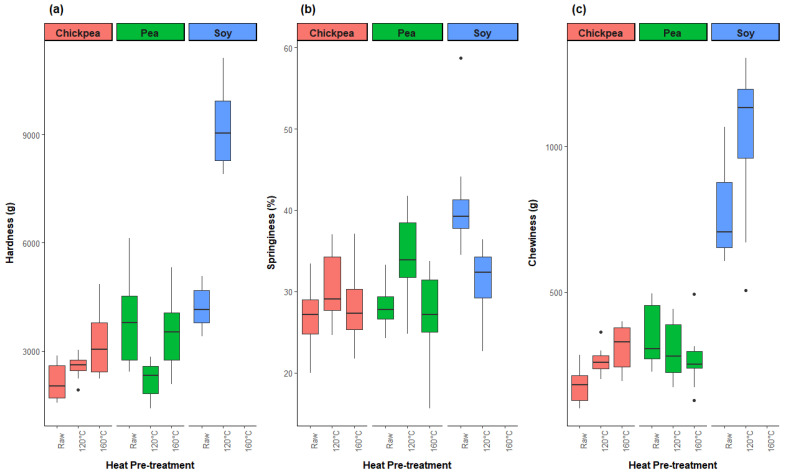
Texture profile analysis, including hardness (**a**), springiness (**b**), and chewiness (**c**) of soy, pea, and chickpea extrudates.

Figure 9 shows that the soy extrudates had significantly higher hardness and chewiness at the 120 °C level compared to any of the pea and chickpea samples. The pea extrudates exhibited the lowest hardness and the highest springiness at the 120 °C level, while the pea and chickpea extrudates demonstrated relatively similar chewiness and hardness across the different pretreatment levels.

### 3.3. Statistical Analysis

The dataset used for the overall statistical analysis comprised 24 samples with 8 predictor variables (powder properties) and 16 output variables (extrudate characteristics), assessed across 3 different pretreatments. The pretreatment levels considered were raw, 120 °C, and 160 °C, except for the extrudates of soy protein at 160 °C due to its processability limitation.

The normality of the data was assessed to determine the appropriateness of the parametric statistical analyses. As illustrated in the Manhattan plot in the Supplementary Materials, −log10 (*p*-value) was plotted to visualize the deviation from normal distribution for each material property. A dashed horizontal line represents the −log10 transformation of the conventional alpha level of 0.05, serving as a threshold for significance, with points above this line indicating a significant departure from normality. This heterogeneity in distributions necessitated the use of both parametric and non-parametric statistical methods for subsequent analyses. A combination of ANOVA and Kruskal–Wallis tests was used to evaluate the significance of the protein source and treatment effects on the various material properties. The analysis of the results table in the Supplementary Materials (Table S1) shows that properties such as extrudate gumminess, chewiness, cohesion, WHC, and lightness were significantly impacted by both the protein source and the treatment (*p* < 0.001), whereas properties such as extrudate hardness and powder peak viscosity were influenced only by the pretreatment level.

The PLS regression model was used to assess how the extrudate properties were associated with the protein powder properties; it is particularly useful when the predictor variables are highly collinear or the number of predictors exceeds the number of observations, both of which were the case in this study. For most properties, the root mean square error of prediction (RMSEP) decreases as the number of components increases, indicating that additional components improve the predictive accuracy of the model. For example, the RMSEP for Hardness decreased from 1.022 to 0.5132 with seven components, indicating an improvement in predictive accuracy with the inclusion of more components. Therefore, the number of components to be considered for the model was selected to be seven. However, it is important to consider that using too many components can lead to overfitting, an overly complex model, and degradation of the generalized predictive performance. The RMSEP values for different response variables vary, indicating that the predictive accuracy of the model differs across extrudate properties. The complete list of RMSEP values for all response variables is provided in the Supplementary Materials.

The loadings for the first two components of the PLS model provide insights into the contribution of each original variable to the components. For the first component, variables such as Aw, L, peak_viscosity, and WAC showed positive loadings, suggesting that this component might represent the hydration-related properties of the ingredients. Conversely, the second component showed a positive correlation with a and peak_viscosity, indicating that this component might represent a combination of color-related properties and viscosity. The coefficients of the PLS model for the two components revealed the influence of each predictor variable on the response variables. For example, peak_viscosity had a strong positive effect on hardness, gumminess, and chewiness, indicating that higher peak viscosities of the powder are associated with increased hardness, gumminess, and chewiness in the extrudate. The root mean square error of prediction (RMSEP) values obtained from cross-validation indicated the model’s predictive performance for each response variable. For instance, the RMSEP for hardness decreased from 1.02 (intercept) to 0.51 with seven components, indicating an improvement in predictive accuracy with the inclusion of more components.

**Figure 10:**
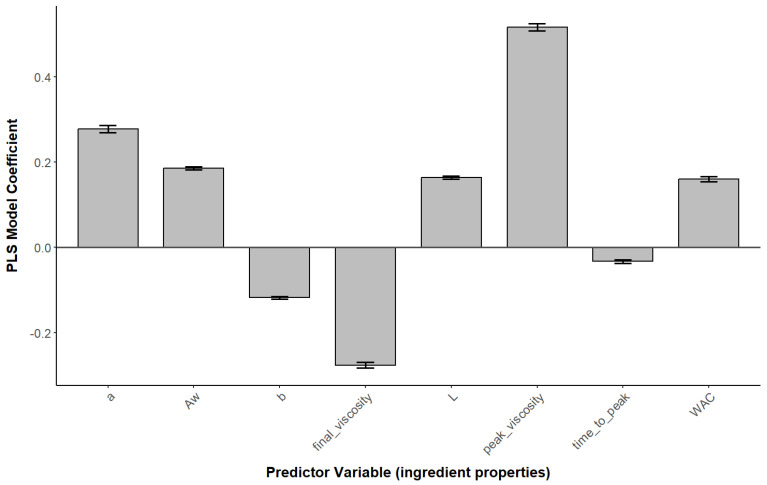
PLS model coefficients for ingredient properties to predict extrudate hardness.

Overall, the PLS regression analysis provided valuable insights into the relationships between powder properties and extrudate properties in plant-based protein ingredients. The model highlighted key variables influencing the textural properties of the extrudate, which can inform the optimization of processing conditions for the production of textured vegetable protein products. To assess the robustness of the PLS regression, a Monte Carlo simulation was performed, which involved repeatedly subsampling the dataset and recalculating the PLS model to observe the variability in the coefficients across multiple iterations. The Monte Carlo analysis, involving 100 simulation iterations, revealed that certain model coefficients exhibited more variability than others. For example, the coefficients related to peak_viscosity and final_viscosity showed relatively stable values across simulations, suggesting that these variables have a consistent impact on the extrudate properties, whereas the coefficients for WAC displayed more variation. The average coefficients obtained from the Monte Carlo simulations were used to summarize the overall effect of each predictor variable on the response variables, as demonstrated for the impact of predictor variables on extrudate hardness in Figure 10.

## 4. Conclusions

This study explored the impact of dry heat pretreatment on the functionality of soy, chickpea, and pea protein ingredients for their subsequent application in texturized vegetable protein (TVP) production via low moisture extrusion. Our findings reveal that pretreatment temperature has a non-linear impact on the functional properties of these proteins. An optimal pretreatment temperature of 120 °C was identified, which enhanced the pasting properties and maintained the water absorption capacity (WAC) of the protein ingredients. The varying responses of different protein sources to heat pretreatment, as evidenced by the DSC results, demonstrate the state of protein denaturation for industrial protein ingredients and the variable potential to modulate functional properties based on preconditioning parameters, such as ingredient pasting properties, water absorption capacity, particle size distribution, and color attributes. The observed increase in extrudate density and darkness when comparing 120 °C to 160 °C pretreatment levels emphasizes the impact of the ingredient protein state, which is related to the balance between protein unfolding and aggregation, on the attributes of low-moisture extrudates. Overall, our research provides insights into the interplay between protein sources, pretreatment conditions, and extrusion outcomes, highlighting the importance of tailored pretreatment for different protein types to control protein denaturation and optimize extrusion outcomes. However, definitive conclusions cannot be drawn on the interplay between these aspects at this stage. Further studies are needed to assess these impacts in relation to consumer acceptance of taste, texture, and overall mouthfeel, to ensure the development of plant-based meat analogues that not only meet functional and textural requirements but also satisfy consumer expectations.

## Figures and Tables

**Table 1:** Composition of commercial protein powders. N/A indicates components not analyzed by the manufacturer.

Component	Soy	Chickpea	Yellow Pea
Protein (%)	93.1	81.4	86.0
Moisture (%)	4.5	7.0	3.0
Fat (%)	0.3	0.4	N/A
Ash (%)	N/A	2.7	N/A
Crude Fiber (%)	N/A	0.7	N/A

**Table 2:** Total color difference (ΔE) between pretreated powder samples and raw powder samples.

Protein	Treatment	ΔE
Chickpea	80	0.63
Chickpea	100	0.55
Chickpea	120	0.35
Chickpea	140	2.19
Chickpea	160	11.00
Pea	80	1.10
Pea	100	0.80
Pea	120	5.21
Pea	140	2.50
Pea	160	8.73
Soy	80	0.72
Soy	100	0.97
Soy	120	0.21
Soy	140	3.23
Soy	160	6.28

**Table 3:** DSC thermal analysis results of onset temperature, peak temperature, and protein denaturation enthalpy (ΔH) for raw and heat pretreated protein powders. The percent reduction in ΔH is in comparison to the raw sample for each protein powder, where 100% indicates complete loss of the protein denaturation peak. * Only one powder was successfully analyzed for the chickpea 120 °C sample.

Protein	Treatment	Onset Temperature (°C)	Peak Temperature (°C)	ΔH (J/g)	Reduction ΔH (%)
Chickpea	Raw	89.5 ± 0.2	107.1 ± 0.8	−0.25 ± 0.25	0
Chickpea	120 °C	92.4 ± 0.0	102.9 ± 0.0	−0.09 *	63.0
Chickpea	160 °C	97.8 ± 2.1	105.1 ± 0.1	−0.07 ± 0.07	73.6
Pea	Raw	79.9 ± 0.4	87.8 ± 1.0	−0.71 ± 0.07	0
Pea	120 °C	78.0 ± 0.3	85.9 ± 0.3	−0.60 ± 0.01	15.3
Pea	160 °C	-	-	0	100
Soy	Raw	-	-	0	-
Soy	120 °C	-	-	0	-
Soy	160 °C	-	-	0	-

**Table 4:** Total color difference (ΔE) from powder to extrudate samples.

Protein	Treatment	ΔE
Chickpea	Raw	8.20
Chickpea	120	8.13
Chickpea	160	11.11
Pea	Raw	19.35
Pea	120	19.63
Pea	160	24.38
Soy	Raw	21.44
Soy	120	23.81

## Data Availability

The original contributions presented in the study are included in the article/Supplementary Material, further inquiries can be directed to the corresponding author.

## Supplementary Materials

The following supporting information can be downloaded at: https://www.mdpi.com/article/10.3390/foods13142168/s1, Figure S1: DSC thermograph of chickpea protein isolate pretreated under different conditions, Figure S2: DSC thermograph of yellow pea protein isolate pretreated under different conditions, Figure S3: DSC thermograph of soy protein isolate pretreated under different conditions, Figure S4: Manhattan plot of normality test results, Figure S5: Partial least squares (PLS) regression coefficients for various response variables in the model, Table S1: Summary of statistical analyses for material properties. This table presents the *p*-values obtained from either the ANOVA or the Kruskal–Wallis test, chosen based on the data distribution for each material property. Significance levels for protein and treatment effects are denoted by asterisks, where ‘***’ indicates *p* < 0.001, ‘**’ indicates *p* < 0.01, ‘*’ indicates *p* < 0.05, and ‘ns’ indicates non-significance.
