# Supplementary material for: Ingredient Functionality of Soy, Chickpea, and Pea Protein before and after Dry Heat Pretreatment and Low Moisture Extrusion
Source: Foods. 2024 Jul 9;13(14):2168. doi: 10.3390/foods13142168 (PMC11276295; doi:10.3390/foods13142168)
Supplement: Supplementary file 1 [file foods-13-02168-s001.zip › foods-3072248-supplementary.pdf]

Supplementary S1

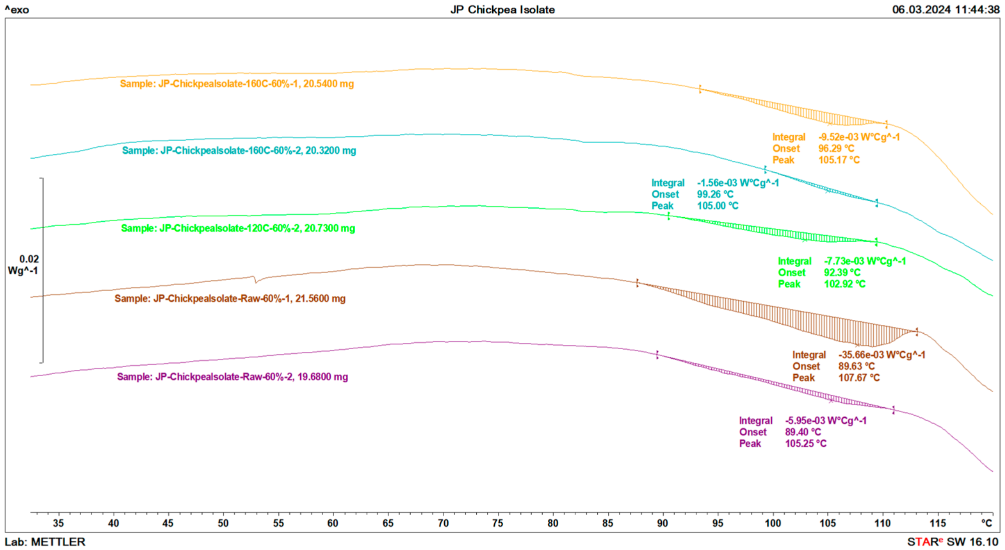

Figure S1. DSC thermograph of chickpea protein isolate pre-treated under different conditions.

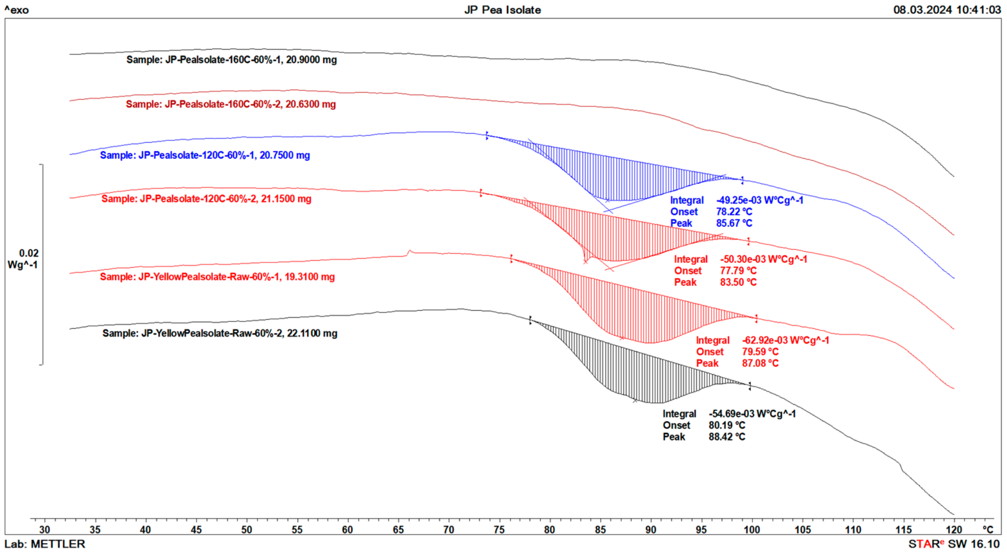

Figure S2. DSC thermograph of yellow pea protein isolate pre-treated under different conditions.

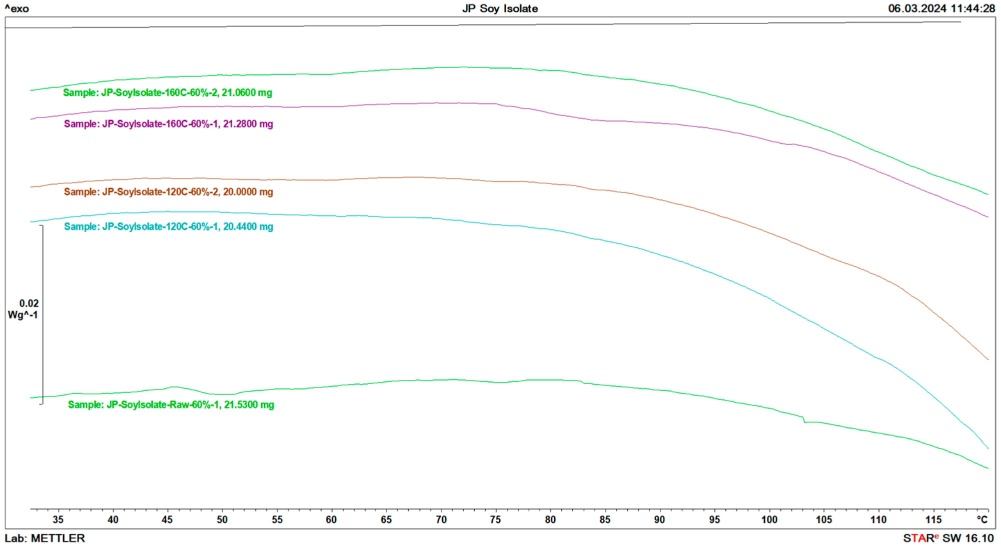

Figure S3. DSC thermograph of soy protein isolate pre-treated under different conditions.

Supplementary S2

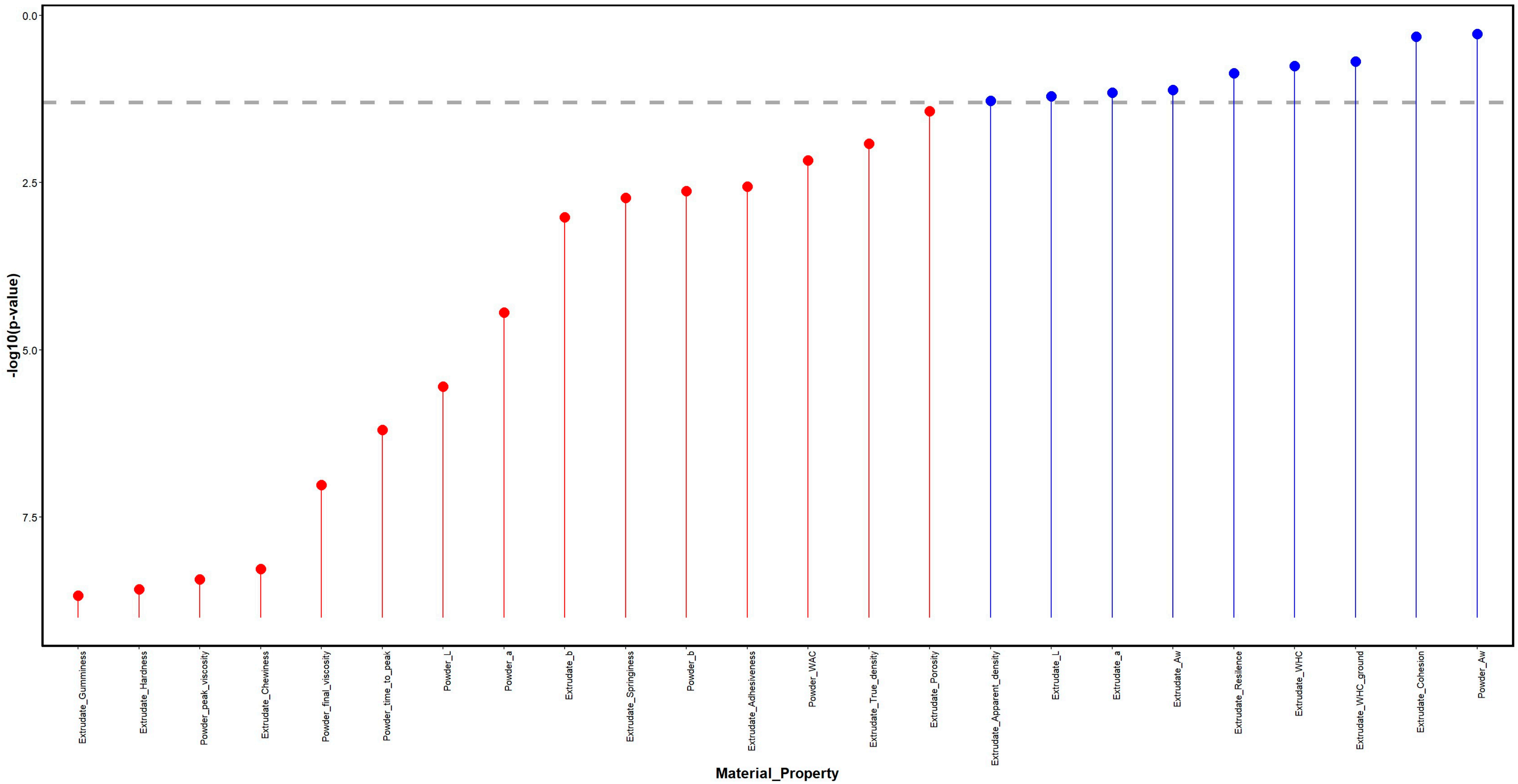

Figure S4. Manhattan plot of normality test results.

Table S1. Summary of statistical analyses for material properties. This table presents the p-values obtained from either the ANOVA or the Kruskal-Wallis test, chosen based on the data distribution for each material property. Significance levels for protein and treatment effects are denoted by asterisks, where '***' indicates p < 0.001, '**' indicates p < 0.01, '*' indicates p < 0.05, and 'ns' indicates non-significance.

Material_Property	P_Value	minus_log10_p_value	Test_Type	Protein_Significance	Treatment_Significance
Extrudate_Gumminess	0.0000	8.6752	Kruskal-Wallis	*** (0)	*** (5e-04)
Extrudate_Hardness	0.0000	8.5796	Kruskal-Wallis	ns (0.9496)	*** (0)
Powder_peak_viscosity	0.0000	8.4326	Kruskal-Wallis	ns (0.0912)	*** (0)
Extrudate_Chewiness	0.0000	8.2709	Kruskal-Wallis	*** (0)	* (0.0156)
Powder_final_viscosity	0.0000	7.0182	Kruskal-Wallis	* (0.0348)	*** (0)
Powder_time_to_peak	0.0000	6.1939	Kruskal-Wallis	*** (0)	** (0.0039)
Powder_L	0.0000	5.5457	Kruskal-Wallis	** (0.0075)	*** (0)
Powder_a	0.0000	4.4392	Kruskal-Wallis	*** (0)	*** (0)
Extrudate_b	0.0010	3.0208	Kruskal-Wallis	*** (0)	ns (0.9982)
Extrudate_Springiness	0.0019	2.7311	Kruskal-Wallis	* (0.0438)	*** (0)
Powder_b	0.0023	2.6297	Kruskal-Wallis	*** (0)	ns (0.5707)
Extrudate_Adhesiveness	0.0028	2.5563	Kruskal-Wallis	*** (0)	ns (0.5099)
Powder_WAC	0.0067	2.1708	Kruskal-Wallis	*** (4e-04)	* (0.0275)
Extrudate_True_density	0.0120	1.9212	Kruskal-Wallis	*** (0)	ns (0.6524)
Extrudate_Porosity	0.0372	1.4299	Kruskal-Wallis	*** (0)	ns (0.1491)
Extrudate_Apparent_density	0.0526	1.2790	ANOVA	*** (1e-04)	ns (0.8474)
Extrudate_L	0.0613	1.2127	ANOVA	*** (0)	*** (2e-04)
Extrudate_a	0.0697	1.1568	ANOVA	*** (3e-04)	ns (0.1913)
Extrudate_Aw	0.0765	1.1162	ANOVA	ns (0.1899)	*** (1e-04)
Extrudate_Resilence	0.1358	0.8670	ANOVA	*** (0)	*** (0)
Extrudate_WHC	0.1735	0.7608	ANOVA	*** (0)	*** (4e-04)
Extrudate_WHC_ground	0.2041	0.6902	ANOVA	*** (1e-04)	ns (0.4896)
Extrudate_Cohesion	0.4801	0.3187	ANOVA	*** (0)	** (0.0011)
Powder_Aw	0.5257	0.2792	ANOVA	*** (0)	ns (0.3531)

Supplementary S3

Partial least squares (PLS) regression was used to investigate the relationship between the properties of powder ingredients and various attributes of the extrudates. PLS regression is a multivariate statistical approach that models the relationship between a set of independent variables (X) and dependent variables (Y). For this study, the *pls* package in R was employed to perform the analysis. Data was compiled for all the ingredients and extrudate samples across all the characterization methods performed. Predictor variables (X) included measured powder properties, while response variables (Y) encompassed a range of textural and other extrudate properties. Prior to modeling, the raw data underwent preprocessing, where the protein treatment and property variables were combined, and the data was filtered, reshaped, and scaled to ensure comparability across scales. The PLS regression models were constructed with a varying number of components to identify the optimal model complexity. Cross-validation was utilized to prevent overfitting and to determine the number of latent variables that provided the best predictive performance, as indicated by the Root Mean Square Error of Prediction (RMSEP). The final model was summarized to assess the importance of each variable in predicting the extrudate properties. The coefficient loadings from the PLSR model provided insights into which powder properties were most influential in determining the extrudate characteristics.

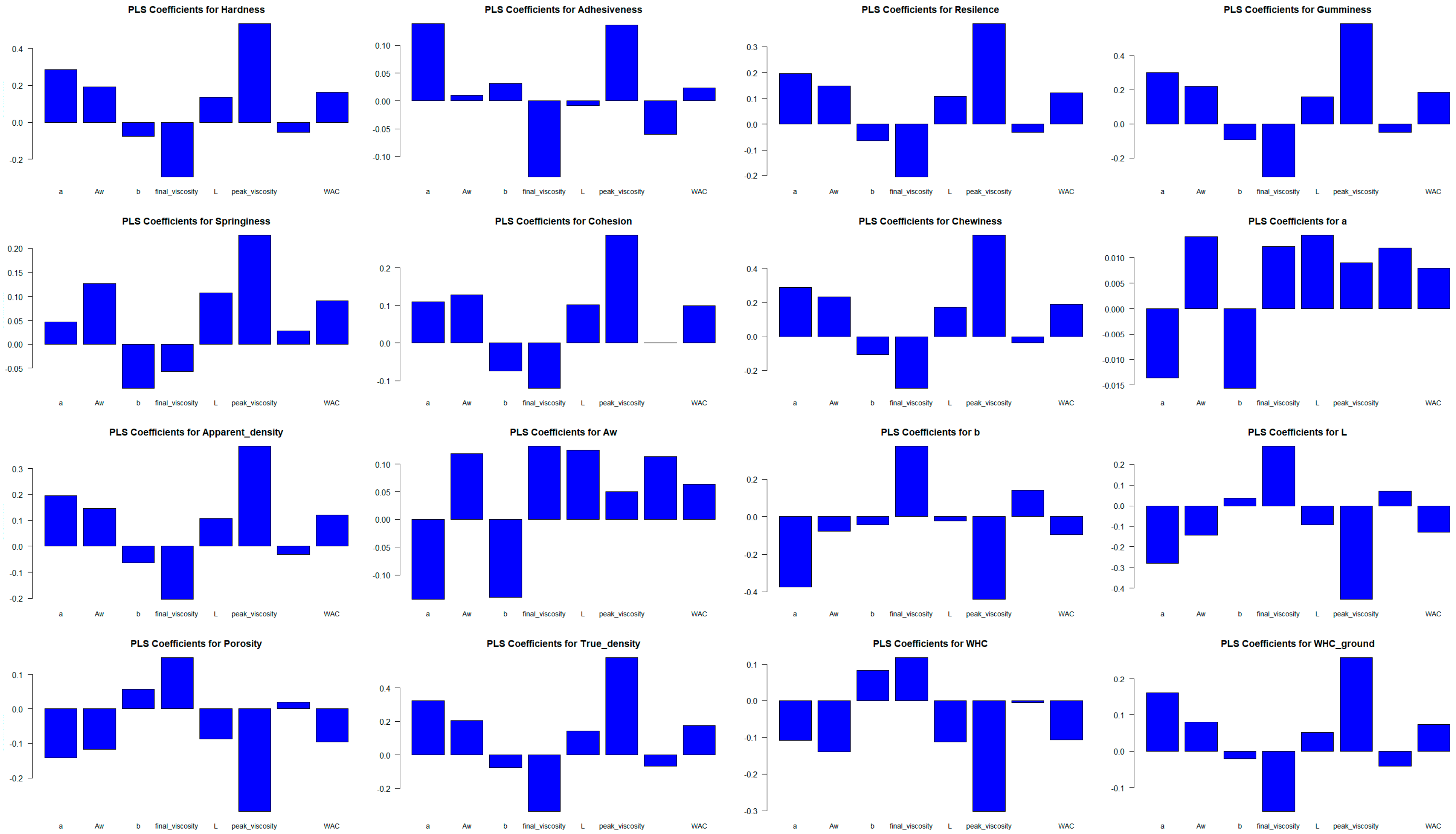

Figure S5. Partial Least Squares (PLS) regression coefficients for various response variables in the model
